# Case Report: Orbital Myositis and Myasthenia Gravis as Symptoms of Immune Reconstitution Inflammatory Syndrome in a Patient With Human Immunodeficiency Virus Infection

**DOI:** 10.3389/fimmu.2020.595068

**Published:** 2020-12-14

**Authors:** Yanli Wang, Ning Zhao, Jun Yang, Ying Wen

**Affiliations:** ^1^ Infectious Diseases Department, The First Affiliated Hospital of China Medical University, Shenyang, China; ^2^ Department of Ophthalmology, The First Affiliated Hospital of China Medical University, Shenyang, China; ^3^ Neurology Department, The First Affiliated Hospital of China Medical University, Shenyang, China

**Keywords:** orbital myositis, myasthenia gravis, acquired immune deficiency syndrome, autoimmune diseases, antiretroviral therapy

## Abstract

We present a case of a 37-year-old man with HIV infection who had been on antiretroviral therapy for one year. He was admitted to our hospital with red and swollen eyes, acute onset progressive exophthalmos, and intermittent diplopia endured for 7 days. His symptoms, exam, and imaging led to a diagnosis of immune reconstitution inflammatory syndrome associated orbital myositis. His symptoms improved considerably after glucocorticoid therapy. Following a reduction in the oral prednisone dose, he re-presented with left ptosis, which rapidly progressed to bilateral ptosis. Diagnostic testing led to the diagnosis of immune mediated myasthenia gravis. Treatment with pyridostigmine bromide, prednisone, and tacrolimus was initiated. One month later, the patient’s symptoms improved significantly. There was a probable association between his symptoms and autoimmune immune reconstitution inflammatory syndrome. This report highlights the importance of recognizing autoimmune disorders in human immunodeficiency virus-infected patients undergoing antiretroviral therapy. Orbital myositis and myasthenia gravis in human immunodeficiency virus-infected patients correlate closely with immunity status following a marked increase in CD4^+^ T cell counts.

## Introduction

Antiretroviral therapy (ART)-induced immune reconstitution is characterized by a rapid reduction in viral load and an increase in CD4^+^ T cell counts. Apart from targeting pathogens, immune reconstitution also targets autoantigens. Immune reconstitution inflammatory syndrome (IRIS)-related autoimmune diseases are uncommon in patients with human immunodeficiency virus (HIV)-infection. This report presents a rare case of a patient with HIV-infection who developed severe orbital myositis as the first IRIS presentation, followed by myasthenia gravis (MG). The sequential occurrence of both conditions in an individual with HIV-infection post-ART has never been reported. Thus, our case emphasizes the importance of recognizing autoimmune disorders as a manifestation of immune reconstitution in patients with HIV-infection undergoing ART.

## Case Description

**Figure 1:**
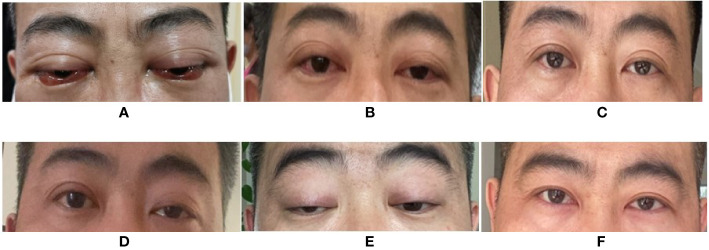
Changes of patient’s eyes’ appearance. **(A)** Eyes’ appearance before therapy of orbital myositis. **(B)** After 3 weeks of treatment, the eye signs improved. **(C)** After 7 weeks of treatment, the eyes’ appearance almost got back to normal. **(D)** New symptoms of left ptosis presented with MG. **(E)** The patient rapidly progressed to obvious bilateral ptosis when MG was diagnosed. **(F)** The patient got a great improvement after one month treatment of pyridostigmine bromide, tacrolimus and prednisone. (Informed consent was obtained).

A 37-year-old Chinese man presented to our hospital in April 2020 with red, swollen eyes, acute worsening exophthalmos, and intermittent diplopia sustained for 7 days. A year earlier, he had been diagnosed with HIV-1 and had started ART [lamivudine (3TC) 300 mg daily, tenofovir disoproxil 300 mg daily, and dolutegravir 50 mg daily] regimen. Following the ocular symptoms, the tenofovir disoproxil and dolutegravir were replaced with lopinavir/ritonavir 400 mg/100 mg twice a day and zidovudine (ZDV) 300 mg twice a day at another hospital one week before this visit. His nadir CD4^+^ T cell count was 242 cells/μl, which increased to 717 cells/μl at the time of examination. His HIV RNA load was less than 20 copies/ml. He was afebrile. An ophthalmologic examination showed proptosis, congested, and edematous conjunctivae, protruding beyond the lid margin, without ptosis. His corneas, fundi, and visual acuity were normal (**Figure 1A**), though his eyeball mobility was limited bilaterally. The patient had no personal or family history of autoimmune disease.

**Figure 2:**
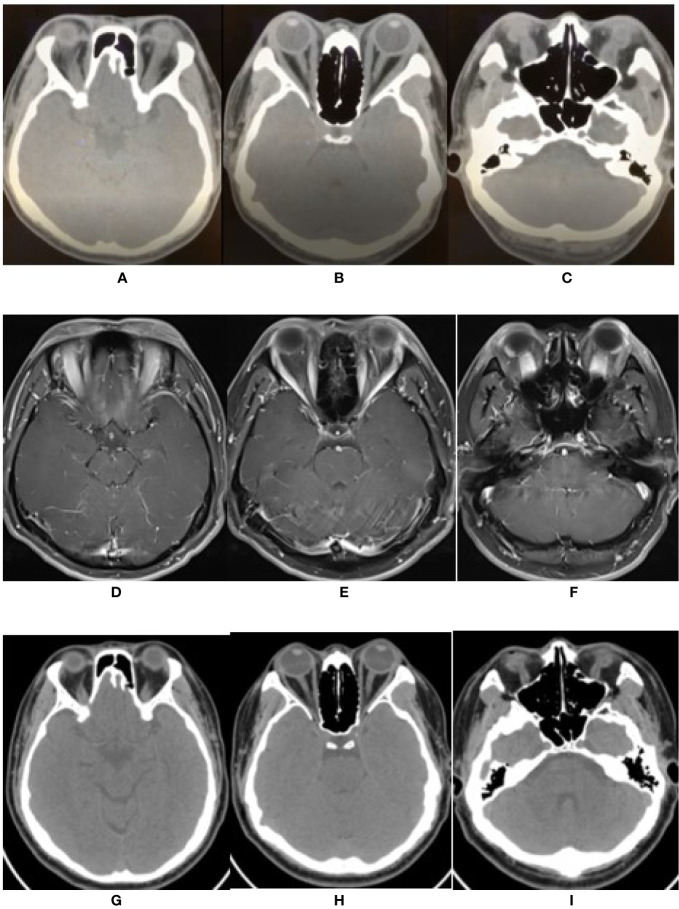
The changes of imaging of extra-ocular muscles. **(A–C)** Orbital CT scan before therapy showed the bilateral ophthalmic muscles including superior rectus, medial and lateral rectus, inferior rectus were obviously thickened. **(D–F)** Enhanced MRI revealed thickened and enhanced multiple extra-ocular muscles including tendinous insertion. **(G–I)** After 3 months of treatment, orbital CT showed bilateral extra-ocular muscles were not thickened.

Orbital computed tomography (CT) confirmed that both eyes were slightly protruding. The extraocular muscles, especially the superior and inferior recti, were enlarged bilaterally (**Figures 2A–C**). The optic nerves were normal. Ocular color ultrasound imaging showed mild, bilateral vitreous opacities and widening of the superior ophthalmic veins. Brain magnetic resonance imaging (MRI) showed no parenchymal abnormality. Contrast-enhanced MRI revealed thickening and increased signal intensity of the extraocular muscles, including the tendinous insertions. Both eyelids and lateral soft tissues showed diffuse swelling, without the lacrimal gland and periorbital fat involvement (**Figures 2D–F**). Aztreonam and clindamycin intravenous antibiotics were administered for 3 days; nonetheless, there was no clinical improvement.

Graves’ orbitopathy (GO) was initially suspected. However, thyroid function tests involving measurements of triiodothyronine; thyroxine, thyroid stimulating hormone, thyroglobulin, thyroid peroxidase, and thyrotropin receptor antibodies as well as color-flow Doppler sonography scan, were normal. Further blood results included the following: serum immunoglobins [IgA, 4.03 g/L (range 0.7–3.8 g/L), IgG, 12.39 g/L (7–17g/L), IgM, 1.89 g/L (0.6–2.5 g/L)] and antibody tests (1:100-positive antinuclear antibody and negative antineutrophil cytoplasmic antibody). The orbital involvement in immunoglobulin G4-related disease (IgG4-RD) was excluded due to normal serum IgG4 levels, absence of eosinophilia (leukocyte 9.39 ×10^9^/L, eosinophilia 0.42 ×10^9^/L), and lack of lacrimal gland involvement. Serum antibody tests for herpes simplex virus, varicella-zoster virus, cytomegalovirus, toxoplasma, and syphilis were negative. Serum Epstein–Barr virus-DNA was absent, and the blood T-SPOT. TB assay to detect T cells primed to mycobacterium tuberculosis (TB) infection was negative. Finally, a diagnosis of IRIS-associated orbital myositis (IRIS-OM) was considered. Dexamethasone (5 mg) was administered intravenously for 5 days, followed by oral prednisone (30 mg) daily. The patient’s proptosis, diplopia, and limited ocular movements improved considerably (**Figures 1B, C**) with an MRI of the extraocular muscles showing reduced swelling (**Figures 2G–I**).

Three months later, when tapering the prednisone, the patient presented with left ptosis, followed immediately by bilateral ptosis and diplopia (**Figures 1D, E**). He complained of facial muscles’ weakness when chewing and weakness of both upper limbs’ muscles while driving. Neurological examination showed bilateral ptosis and disconjugate eye movements without detectable facial or upper limb muscles’ weakness. His sensation, coordination, and deep tendon reflexes were normal. A neostigmine challenge test was positive. Further blood tests showed a positive result for the ryanodine receptor (RyR). Acetylcholine receptor (AChR), muscle-specific tyrosine kinase (MuSK), and titin receptor antibodies were negative. Creatine kinase levels were within normal limits (105 U/L; normal range 39**–**308 U/L). A chest CT scan results excluded thymus abnormalities. Routine nerve conduction studies of the right facial nerve, left ulnar nerve, and left median nerve were normal. Low frequency and high frequency repetitive nerve stimulation using the standard approach was used to evaluate the left ulnar nerve and right facial nerve. The left ulnar nerve demonstrated normal responses, whereas the right facial nerve demonstrated 15% decrement in amplitude at low frequencies without enhancement at high frequencies. Single fiber electromyography (EMG) and needle EMG were not performed. Based on the outlined findings, the patient was diagnosed with probable IRIS-associated MG. He was treated with oral pyridostigmine bromide, 60 mg three times per day; tacrolimus, 1 mg twice a day; prednisone, 30 mg once a day for 2 weeks, followed by 20 mg daily for 3 months. One month later, the patient showed considerable improvement (**Figure 1F**); facial and upper limb muscle weakness improved more rapidly than the ptosis. At the time of this report, the serum creatine kinase was 33 U/L (39**–**308 U/L), the CD4^+^ T cell count had dropped to 369 cells/μl, and the HIV RNA load was undetectable. He had no complaints with the current pyridostigmine bromide (60 mg daily), prednisone (20 mg daily), and tacrolimus (2 mg daily) treatment regimen, together with ART (**Table 1**).

**Table 1:** Table showcasing a timeline with relevant data.

	Diagnosed with AIDS	HAART for 7 months	Onset of orbital myositis (HAART for 12 months)	Onset of MG (HAART for 15 months)	nowadays (HAART for 18 months)
CD4 cell count(/μl)	242	299	717	No detection	369
HIVRNA	No detection	less than 20 copies/ml	less than 20 copies/ml	less than 20 copies/ml	less than 20 copies/ml
CK (39–308 U/L)	No detection	No detection	No detection	105 U/L	33 U/L
symptoms	Found AIDS in physical examination. No symptoms and opportunistic infection	No eyes symptoms and muscles weakness	Swollen of both eyes, acute worsening exophthalmos and intermittent diplopia	Bilateral ptosis, chewing weakness and mild both upper limbs muscles weakness	No eyes symptoms. No chewing weakness and upper limbs muscles weakness
HAART	3TC, TDF, DTG	3TC, TDF, DTG	immediately changed to 3TC, LPV/RTV, ZDV	changed to 3TC, LPV/RTV, ZDV for 3 months	3TC, LPV/RTV, ZDV
medication	None	None	Dexamethasone 5mg, 5 days. followed by prednisone 30mg per day and tapering	When prednisone was tapered to 10 mg, MG symptom was developed. pyridostigmine bromide 60 mg three times a day, tacrolimus 1 mg twice a day, prednisone 30 mg once a day for two weeks, followed by 20mg once a day for three months	Pyridostigmine bromide 60 mg per day, prednisone 20 mg per day tacrolimus 2mg per day

## Discussion

We have presented a rare case of a patient with HIV infection who developed severe orbital myositis as the first IRIS presentation, followed by MG. Although in most cases, IRIS appears during the first 3 months after the initiation of ART (‘early IRIS’), there could be a later occurrence, generally 3–12 months (‘late IRIS’) after ART initiation (1). A few cases even occurred as late as 4 years from the initial treatment (2). This patient met the late IRIS criteria, such as presentation of new symptoms and signs at 12 months, achieving an undetectable HIVRNA and obvious increment in CD4 T^+^ cells count after ART especially recent half year. The pathogenesis of early IRIS is probably caused by memory CD4^+^ T cells, while late IRIS could be the result of the second phase of CD4^+^ T cells increase as naive CD4^+^ T cells migrate from the thymus.

The initial manifestations of the patient were an acute onset of inflammation of the extraocular muscles and proptosis. The reported incidence of proptosis in patients with HIV-infection with ocular diseases is 2.5% (3). Infections and tumor development are the most common etiologies of orbital involvement in acquired immunodeficiency syndromes of any form (4). GO (5) and IgG4-RD are common inflammatory conditions that affect the orbital tissue. In this case, the clinical manifestations, imaging features, and rapid glucocorticoid response made orbital myositis the most likely diagnosis. Orbital myositis is an autoimmune inflammatory process that primarily involves the extraocular muscles and could be excluded in a diagnosis once neoplasm, primary infection, and systemic disorders, have been ruled out (6, 7). Unlike GO, orbital myositis is the term used when the extraocular muscles are the major site of inflammation and constitutes a distinct subset of nonspecific orbital inflammation. Nonspecific orbital myositis is usually unilateral, with the bilateral forms making up 10–20% of all cases (8). It is characterized by a sudden onset of periocular swelling, pain and redness of the eyelids, proptosis, and ptosis (7). Steroid treatment is effective in one-third of cases (7, 9) though spontaneous remission is possible. If steroid therapy fails or recurrence is observed, a biopsy is recommended for further tests (10). Our patient did not undergo a biopsy because of the rapid response to glucocorticoid treatment. Orbital myositis could be the first presenting symptom of HIV infection (11). The orbital soft tissues disease involved in IRIS are rarely reported. Up to now, only two cases were reported in HIV-infected patients. One was a 15-year-old girl with HIV infection undergoing ART for 9 years, who presented orbital myositis with several relapses and remissions (7). Another was reported in a 60-year-old man with HIV infection, who had two orbital inflammation episodes, following CD4^+^ T cell count improvement with ART (12).

Although GO is the most common reason for inflammation of orbital tissues (8, 13), ocular involvement in patients with HIV who have thyroid diseases is uncommon (14). Most patients with GO have abnormal thyroid function tests. Approximately 1–2% of patients with GO do not have clinically overt thyroid dysfunction, although they almost always display evidence of thyroid autoimmunity, including abnormal levels of thyroglobulin thyroid peroxidase and thyrotropin receptor antibodies (8). From their origins to their insertions, the MRI findings of enlarged and edematous ocular muscles could distinguish orbital myositis from GO. Typically, among GO individuals, usually more than one muscle is affected, and the tendinous insertion is not involved (8). Hence, GO could be an IRIS-related unmasking presentation in patients with HIV infection post-ART (15). In IgG4-RD, the most frequent orbital tissue-involved sites are the lacrimal glands and bilateral ocular muscles (7). Although IgG4-related lymphadenopathy has been reported in the initial presentation of HIV infection (16), the orbital disease caused by IgG4-RD has never been reported in patients with HIV infection. The typical manifestations of an orbital lymphoma are diplopia, proptosis, and palpebral ptosis, with a palpable mass in the orbit’s anterior region (8). Imaging, ocular fundus examination, and related blood tests could aid in excluding any infectious, vasculitis, and neoplastic processes (7).

MG is an autoimmune disease of neuromuscular transmission, and is primarily mediated by autoantibodies located in the postsynaptic membrane (17). Anti-AChR antibodies bind to the neuromuscular junction, activate complement, and accelerate AChR destruction, leading to failure of neuromuscular transmission and myasthenic symptoms (17). HIV infection itself is responsible for triggering autoimmune diseases through molecular mimicry (18), which improves with progressive immunosuppression (19). Only a few among the several reported cases of patients with HIV infection presented with an unmasking of MG post-ART (**Table 2**) (19–24), were patients had highly elevated CD4^+^ T cell counts at the time of MG onset. Among the seven reported cases (including the present case), four were female and three were males, all aged between 21 and 71 years who presented MG between 12 months and 60 months after ART was started. Immunosuppressive agents were used in most cases, and the short and mid-term prognoses were good. Although patients with HIV infection who also have MG have been reported to have positive anti-AChR or anti-MuSK antibodies (25), the presence of RyR antibodies has not been reported previously. RyR is one of the antigens that activate Ca^2+^ release channel of the sarcoplasmic reticulum, its RyR antibody is prevalent in thymoma-MG patients who show a good response to tacrolimus (26). Individuals with titin and RyR antibodies might need more active immunosuppressive therapy, while those with RyR antibodies have lower mean daily dosage requirements of pyridostigmine (27). Most RyR antibody-positive patients also have positive AChR antibodies (28). The reported AChR, MuSK, titin, and RyR antibody levels among adult Chinese patients with MG are 82.2, 2.3, 28.4, and 23.8%, respectively (28). The existence of RyR antibodies in AChR antibody-negative patients has been reported mainly in East Asian populations but rarely among Caucasians (28). For patients without positive AChR or MuSK antibodies test, confirmation with at least 10% decrease at the repetitive low frequency stimulation to related nerves is crucial (29). The muscle involvement in individuals with HIV-infection should be differentiated from myopathy, especially ZDV myopathy; long-term ZDV therapy (3**–**21 months) can cause toxic mitochondrial myopathy (30–32). When orbital myositis occurred in this case, the patient’s new ART regimen of 3TC, lopinavir/ritonavir, and ZDV were prescribed to replace the previous regimen of tenofovir disoproxil, 3TC, and dolutegravir. At the onset of MG, the patient had used ZDV for three months. ZDV-induced myopathy was not considered because of the patient’s normal serum creatine kinase level a remarkable improvement in the condition without discontinuation of ZDV.

**Table 2:** Summary of case reports on HIV and MG after ART. M, male; F, female; NM, not mentioned; EFV, Efavirenz; DDI, di-deoxyinosine.

Case	Age	Gender	Thymic hyperplasia	ART and CD4 cell count (/μL)	CD4 cell count (/μL)	Antibody	ART of MG onset	MG Treatment
Kuntzer T et al. (19)	21	F	NM	AZT, 3TC, EFV 2 years, then AZT was replaced by TDF for 8 months, CD4 increased rapidly from 244 to 575.	From 244 to 575.	MUSK+	TDF, 3TC, EFV	Bromopyrimidine, prednisone, thymectomy, gamma globulin, plasma exchange, mycophenolate mofetil, rituximab.
Sherpa et al. (20)	44	F	No abnormality	He stopped ART himself. ART was treated again for 2 years. CD4 increased from 53 to 383.	From 53 to 383.	MUSK+	Emtricitabine, TDF, RTV	Gamma globulin, bromopyrimethamine, azathioprine
Ragunathan et al. (21)	39	F	NM	Salvage therapy 12 months, CD4 increased from 100 to 520.	From 100 to 520.	MUSK+	TDF/emtricitabine, Darunavir, raltegravir	Prednisone, azathioprine, plasma exchanges.
Knopf and Menkes (22)	27	F	thymic hyperplasia	3 years, CD4 increased from 213 to 432.	From 213 to 432.	AchR+	TDFemtricitabine, EFV	Bromopyrimethamine, prednisone, azathioprine, thymectomy.
Saadat and Kaminski (23)	71	M	NM	AZT and 3TC were used for 1 year, combined with ritonavir for 3 weeks CD4 is 290 now (not mentioned before)	CD4 is 290 now (NM before)	AchR-	3TC, DDI	Bromopyrimidine
Kurokawa et al. (24)	58	M	No abnormality	ART 5 years	NM	MUSK+	NM	Bromopyrimethamine, prednisone, cyclosporine.
Our patient	37	M	No abnormality	ART 1 year, CD4 increased from 240 to 717.	From 240 to 717.	RyR+	3TC, LPV/RTV, ZDV	Prednisone, bromopyrimidine, tacrolimus.

Although MG coexists with a second autoimmune disease in 15% of cases (33), the sequential appearance of two autoimmune diseases in an individual with HIV infection post-ART is rare. Autoimmunity might result from a disruption in peripheral tolerance in a genetically susceptible host (18). The main clinical implication of this case is that orbital myositis and MG are closely correlated with immune status in patients with HIV-infection when CD4^+^ T cell counts increase markedly following ART. Attention should be paid to an autoimmune IRIS, which is defined as an exacerbation or the first presentation of autoimmune disease following ART initiation. Notably, the mean interval between the introduction of ART and the onset of autoimmune disease is much longer (several months) than that reported for disorders of infectious origin (a few weeks). Autoimmune IRIS may have a better prognosis than a non-IRIS autoimmune disease. Further study and long-term follow-up are needed to clarify and verify the findings of this case. Since misdiagnosis is common, it is essential to highlight cooperation among multidisciplinary teams, to enable accurate diagnosis and effective treatment.

## Data Availability Statement

The raw data supporting the conclusions of this article will be made available by the authors, without undue reservation.

## Ethics Statement

Written informed consent was obtained from the individual(s) for the publication of any potentially identifiable images or data included in this article.

## Author Contributions

YLW: article writing. NZ, JY, YW: article revising. All authors contributed to the article and approved the submitted version.

## Funding

2019 China Medical University Double First Class Construction Infectious Disease AIDS diagnosis and treatment. Fund Number: 3110119068.

## Conflict of Interest

The authors declare that the research was conducted in the absence of any commercial or financial relationships that could be construed as a potential conflict of interest.

## Abbreviations

MG, myasthenia gravis; HIV, human immunodeficiency virus; ART, antiretroviral therapy; GO, Grave’s orbitopathy; IRIS, immune reconstitution inflammatory syndrome; ZDV, zidovudine; CT, computed tomography; MRI, magnetic resonance imaging; EMG, electromyography; IgG4-RD, immunoglobulin G4-related disease; AChR, acetylcholine receptor; MuSK, muscle-specific receptor tyrosine kinase; RyR, ryanodine receptor.
